# Thin film deposition of metal oxides in resistance switching devices: electrode material dependence of resistance switching in manganite films

**DOI:** 10.1186/1556-276X-8-76

**Published:** 2013-02-15

**Authors:** Toshihiro Nakamura, Kohei Homma, Kunihide Tachibana

**Affiliations:** 1 Department of Electronic Science and Engineering, Kyoto University, Kyotodaigaku-Katsura, Nishikyo-ku, Kyoto 615-8510, Japan

**Keywords:** Resistance switching, ReRAM, Manganite, PCMO, Impedance spectroscopy

## Abstract

The electric-pulse-induced resistance switching in layered structures composed of polycrystalline Pr_1−*x*_Ca_*x*_MnO_3_ (PCMO) sandwiched between Pt bottom electrode and top electrodes of various metals (metal/PCMO/Pt) was studied by direct current current–voltage (*I*-*V*) measurements and alternating current impedance spectroscopy. The *I*-*V* characteristics showed nonlinear, asymmetric, and hysteretic behavior in PCMO-based devices with top electrode of Al, Ni, and Ag, while no hysteretic behavior was observed in Au/PCMO/Pt devices. The PCMO-based devices with hysteretic *I*-*V* curves exhibited an electric-pulse-induced resistance switching between high and low resistance states. Impedance spectroscopy was employed to study the origin of the resistance switching. From comparison of the impedance spectra between the high and low resistance states, the resistance switching in the PCMO-based devices was mainly due to the resistance change in the interface between the film and the electrode. The electronic properties of the devices showed stronger correlation with the oxidation Gibbs free energy than with the work function of the electrode metal, which suggests that the interface impedance is due to an interfacial oxide layer of the electrode metal. The interface component observed by impedance spectroscopy in the Al/PCMO/Pt device might be due to Al oxide layer formed by oxidation of Al top electrode. It is considered that the interfacial oxide layer plays a dominant role in the bipolar resistance switching in manganite film-based devices.

## Background

Recently, a large resistance change by the application of an electric pulse was observed at room temperature in metal oxides such as Pr_1−*x*_Ca_*x*_MnO_3_ (PCMO) [1-31]. This effect provides a possibility of a next-generation nonvolatile memory, called resistance random access memory (ReRAM). ReRAM is highly expected to replace conventional flash memory due to its low power consumption, small bit cell size, and fast switching speed. The underlying mechanism of the resistance switching behavior is still poorly understood, although there have been various proposed models of the resistance switching mechanism such as formation and rupture of conductive filament paths [3,4], field-induced electrochemical migration such as oxygen vacancy creation/diffusion [5,6], alteration of the width and/or height of a Schottky-like barrier by trapped charge carriers in the interface states [7], trap-controlled space-charge-limited current [8-12], injecting electrons into and extracting electrons from the interface [13], and oxidation/reduction reaction at the interface [14-20]. It was also reported that the resistance switching is significantly dependent on electrode materials in the ReRAM devices [14,18,21-26]. The precise identity of the switching location where resistance change mainly occurs has not been revealed. The comprehensive understanding for the origin of the resistance switching is required to meet the requirement for the next-generation nonvolatile memory application.

Impedance spectroscopy is a useful technique for characterizing the resistance switching in metal oxide films, which indicates whether the overall resistance of the device is dominated by a bulk, grain boundary, or interface component [30-39]. In this work, the resistance switching mechanism in PCMO-based devices was investigated by impedance spectroscopy. In order to study the resistance switching mechanism in the PCMO-based devices, the frequency response of complex impedance was measured in the PCMO-based devices with various metal electrodes. Based on impedance spectral data, the electrode material dependence of the resistance switching in the PCMO-based devices was discussed by correlating with the standard Gibbs free energy of the formation of metal oxides and the work function of each electrode metal.

## Methods

Polycrystalline PCMO films were deposited on prefabricated Pt/SiO_2_/Si substrates by radio-frequency (rf) magnetron sputtering with a Pr_0.7_Ca_0.3_MnO_3−*δ*_ target. The base pressure was 1 × 10^−6^ Torr. Before the deposition, the target was presputtered for 30 min to obtain a clean target surface. A mixture of Ar and O_2_ gases with 25% oxygen content was used for the sputter deposition. The process pressure was controlled at 20 mTorr. The rf power was 80 W. The substrate temperature was 450°C. The film thickness was obtained by cross-sectional scanning electron microscopy. All films were about 100 nm thick.

In order to measure the electrical properties of the deposited films, we prepared layered structures composed of PCMO sandwiched between a Pt bottom electrode and top electrodes. Four kinds of metallic electrodes such as Al, Ni, Ag, and Au with a thickness of about 200 nm and a circle area with a diameter of 500 μm were deposited on top of the films by thermal evaporation. The resistance of metal/PCMO/Pt junctions was evaluated by three techniques: (1) current–voltage (*I*-*V*) characteristics, (2) resistance measurements after pulsed voltage application, and (3) Cole-Cole plots by impedance spectroscopy. The positive voltage is defined as the current flows from the top electrode to the PCMO film, and the negative bias was defined by the opposite direction. The resistance switching of the PCMO films was measured by applying a single positive electric pulse and a single negative electric pulse alternately to the top electrode. The width of the electrical pulse was 500 ns. The resistance values were read out at 0.1 V after each pulse. Impedance spectroscopy was performed in the frequency range of 100 Hz to 5 MHz. The oscillatory amplitude for the impedance measurements was 50 mV.

## Results and discussion

**Figure 1:**
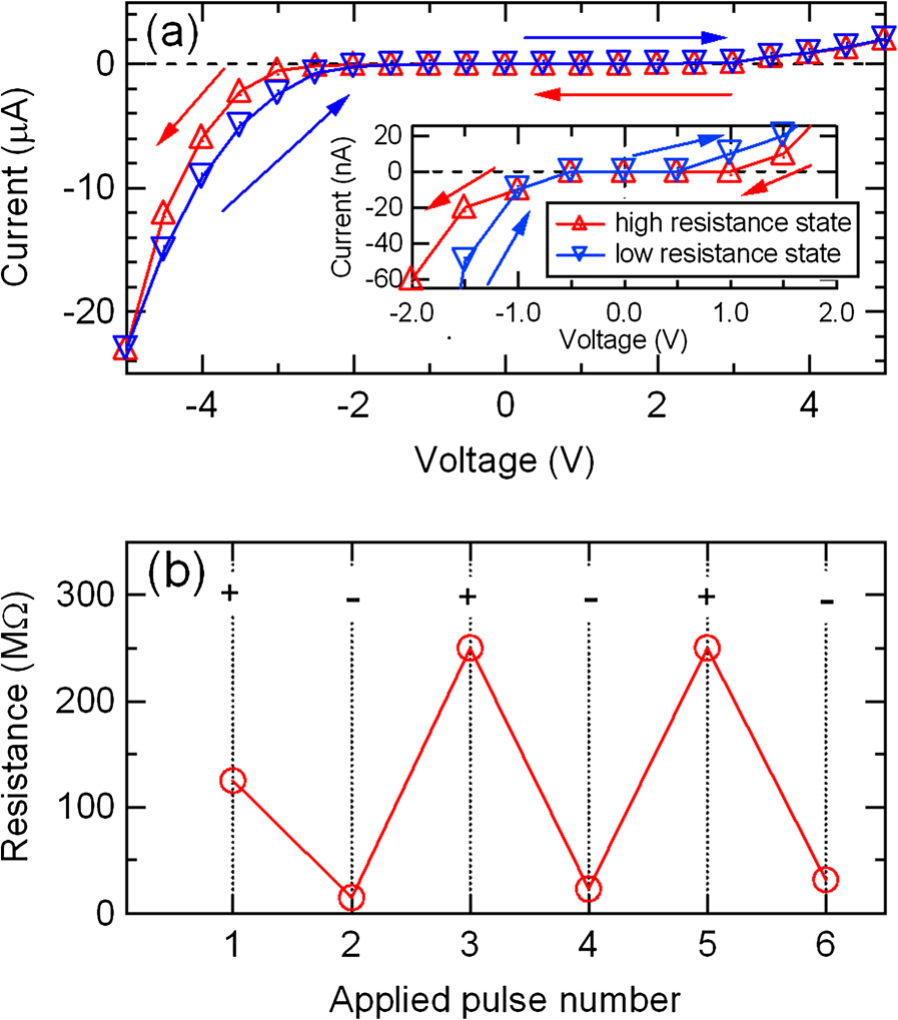
***I*-*V *curves and resistance switching behavior of the Al/PCMO/Pt device.** (**a**) *I*-*V* curves of the Al/PCMO/Pt device. The inset magnifies the behavior near the origin. (**b**) Resistance switching behavior of the Al/PCMO/Pt device.

The *I*-*V* characteristics and resistance switching behaviors of the PCMO-based devices with various kinds of electrode metals were studied by direct current (dc) voltage sweep measurements to evaluate the electrode material dependence of the memory effects. Figure 1a shows the *I*-*V* characteristic of the Al/PCMO/Pt device. The inset magnifies the behavior near the origin. The Al/PCMO/Pt device has nonlinear and asymmetric *I*-*V* relations with hysteresis loops, resulting in resistance memory effect with high and low resistance states during the forward and backward sweeping of the voltage. By increasing the negative voltages, the switching from the high resistance state to the low resistance state occurred. Subsequently, an opposite process was observed by sweeping the voltage reversely to positive values. The resistance change of the PCMO films was measured by applying electric pulses. Figure 1b shows the resistance switching in the Al/PCMO/Pt device. The pulse amplitude was 8 V. The positive or negative pulse reversibly switched the resistance of the PCMO films between the high resistance state and the low resistance state; the nonvolatile switching was achieved.

**Figure 2:**
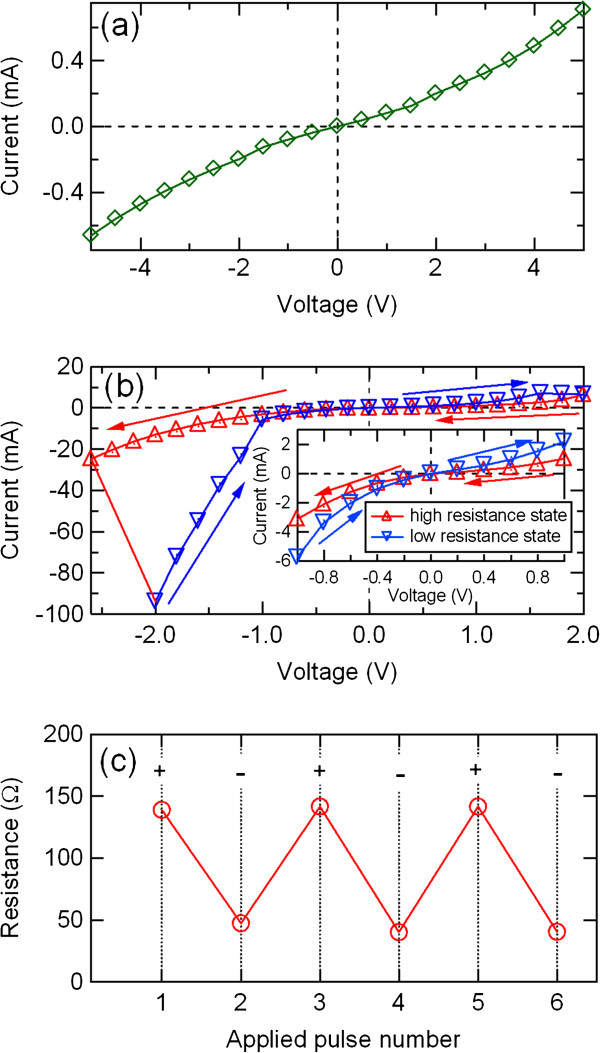
***I*-*V *curves and resistance switching behavior of the Ni/PCMO/Pt device. ***I*-*V* curves in the (**a**) initial state and (**b**) high and low resistance states of the Ni/PCMO/Pt device. The inset magnifies the behavior near the origin. (**c**) Resistance switching behavior of the Ni/PCMO/Pt device.

Figure 2a shows *I*-*V* characteristics in the initial state of the Ni/PCMO/Pt device. The *I*-*V* characteristics exhibited no hysteretic behavior. After adding an electric pulse of 5 V, however, the resistance of the device was decreased, and a hysteretic behavior shown in Figure 2b was observed. An increase in the negative voltages switched the high resistance state to the low resistance state with a negative differential resistance. Figure 2c shows the resistance switching in the Ni/PCMO/Pt device. The amplitude of the applied pulses was 5 V. The switching from the high resistance state to the low resistance state occurred.

**Figure 3:**
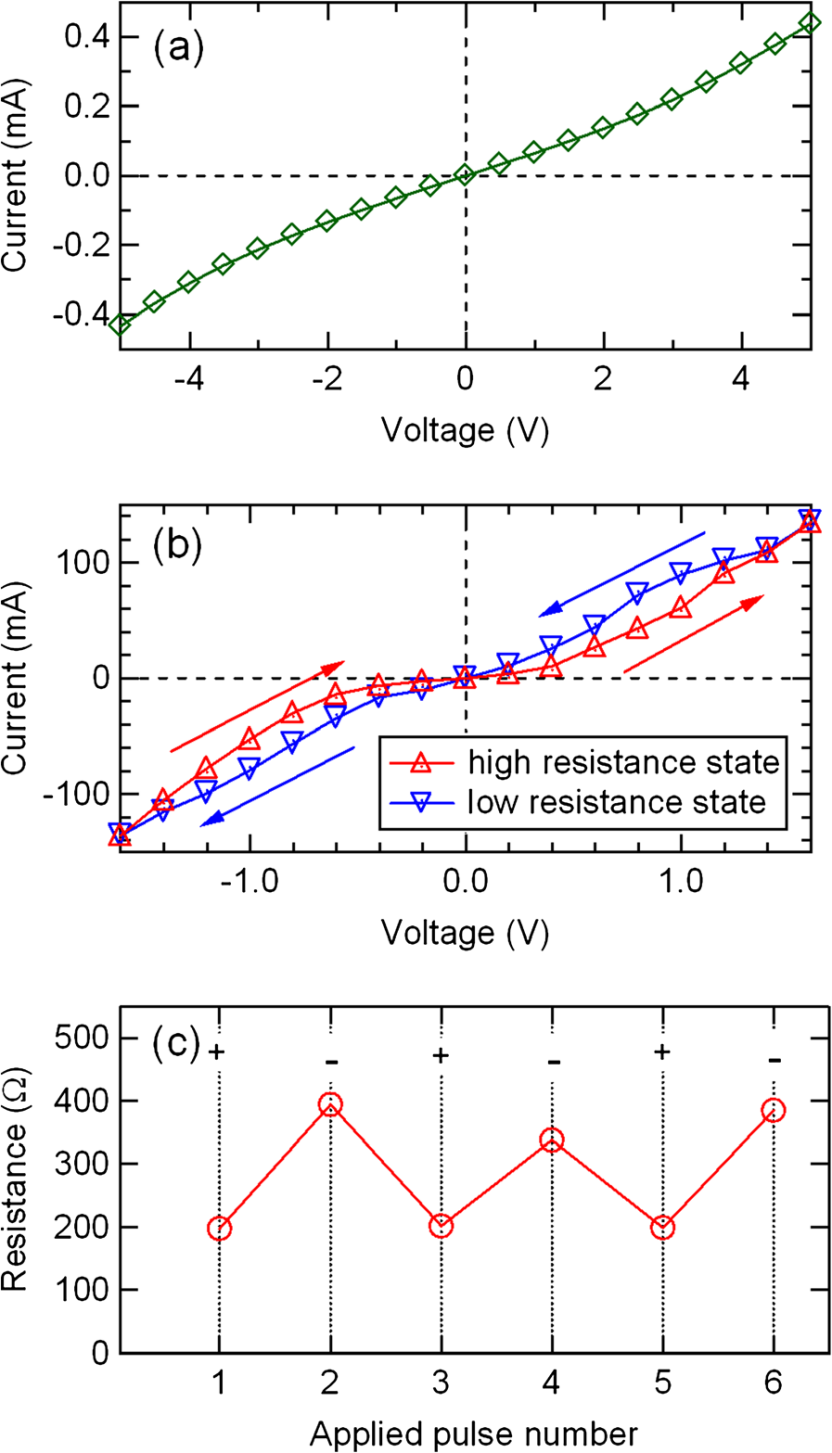
***I*-*V *curves and resistance switching behavior of the Ag/PCMO/Pt device. ***I*-*V* curves in the (**a**) initial state and (**b**) high and low resistance states of the Ag/PCMO/Pt device. (**c**) Resistance switching behavior of the Ag/PCMO/Pt device.

Figure 3a shows *I*-*V* characteristics in the initial state of the Ag/PCMO/Pt device. The *I*-*V* hysteresis was absent as well as the initial state of the Ni/PCMO/Pt device. After adding an electric pulse of 10 V, however, the resistance of the device was decreased, and a hysteretic behavior shown in Figure 3b was observed. Increasing the negative voltages switched the low resistance state to the high resistance state. The Ag/PCMO/Pt device showed an opposite switching direction to the Al/PCMO/Pt and Ni/PCMO/Pt devices in the *I*-*V* characteristics. Figure 3c shows the resistance switching in the Ag/PCMO/Pt device. The pulse amplitude was 10 V. The switching polarity of the Ag/PCMO/Pt device was opposite to that of the Al/PCMO/Pt and Ni/PCMO/Pt devices. This corresponds to the opposite polarity dependence in the *I*-*V* characteristics.

**Figure 4:**
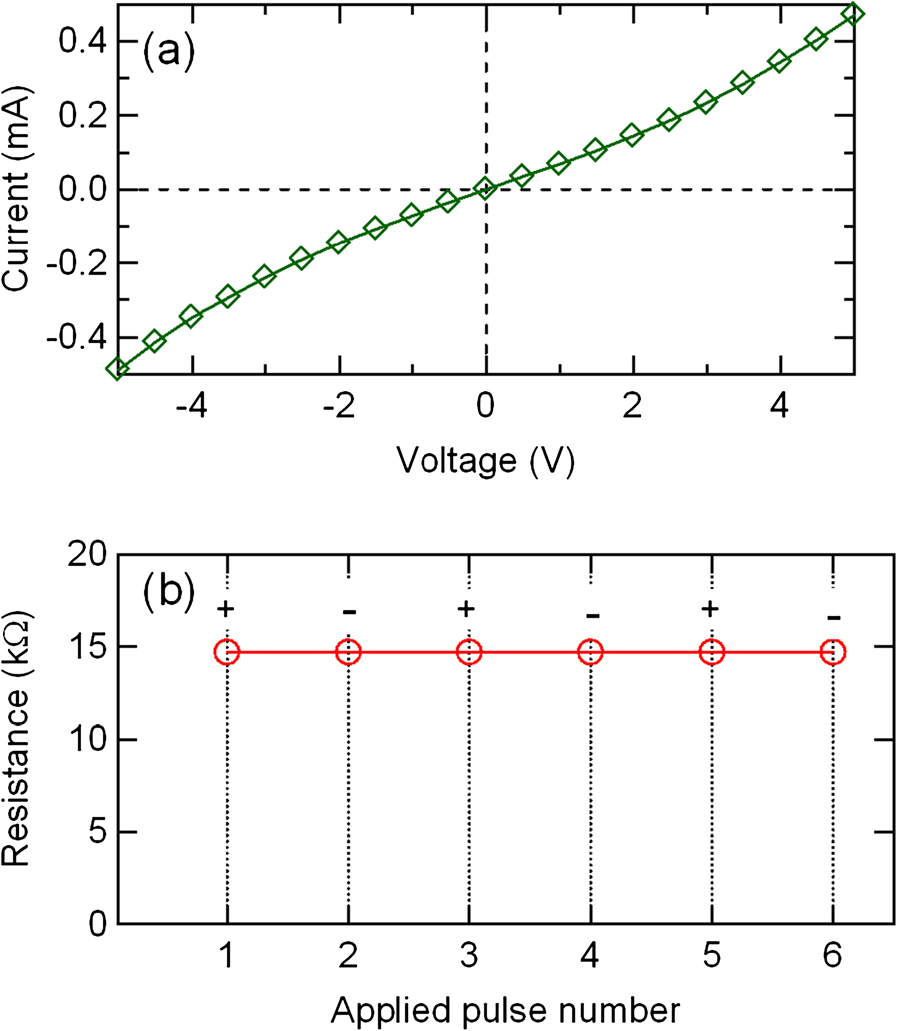
***I*-*V *curve and resistance switching behavior of the Au/PCMO/Pt device.** (**a**) *I*-*V* curve of the Au/PCMO/Pt device. (**b**) Resistance switching behavior of the Au/PCMO/Pt device.

Figure 4a shows *I*-*V* characteristics in the initial state of the Au/PCMO/Pt device. The *I*-*V* characteristics exhibited no hysteretic behavior. Even after adding an electric pulse of 10 V, nonswitching behavior was observed in the *I*-*V* characteristics. Figure 4b shows the behavior of the resistance in the Au/PCMO/Pt device. The pulse amplitude was 10 V. No significant resistance change was observed. This corresponds to the nonswitching *I*-*V* characteristics.

**Figure 5:**
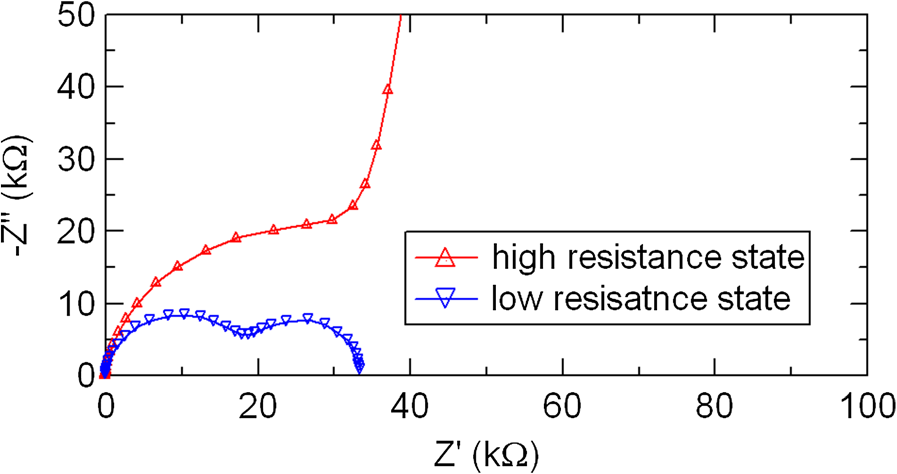
**Impedance spectra of the high and low resistance states in the Al/PCMO/Pt device.** The solid line connects experimental data points.

In order to study the resistance switching mechanism in the PCMO-based devices, the frequency response of complex impedance of the PCMO-based devices was measured. Impedance spectroscopy indicates whether the overall resistance of the device is dominated by a bulk or interface component. We investigated the resistance switching behavior by comparing impedance spectra between high and low resistance states. Figure 5 shows impedance spectra of the Al/PCMO/Pt device. Two semicircular arcs were observed in the Cole-Cole plot. The semicircular arcs in the high and low frequency regions are assigned to the bulk and interface components, respectively [32]. The decrease in the diameters of both semicircular arcs was observed by switching from the high to low resistance states. The switching from the low resistance state to the high resistance state doubled the bulk impedance, while the interface impedance increased about 60 times simultaneously. The change in the interface component was much larger than that in the bulk component.

**Figure 6:**
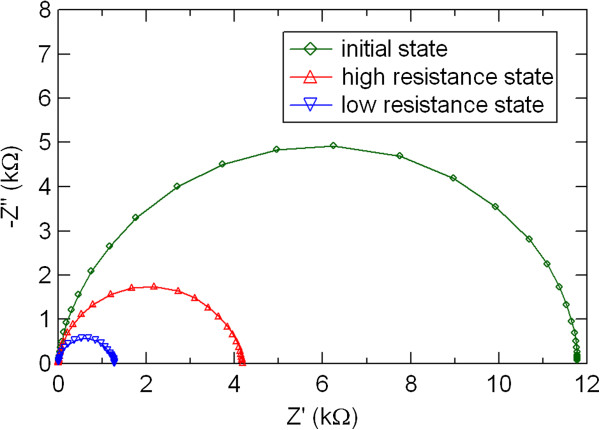
**Impedance spectra of the initial, high resistance, and low resistance states in the Ni/PCMO/Pt device.** The solid line connects experimental data points.

Figure 6 shows impedance spectra of the initial, high resistance, and low resistance states in the Ni/PCMO/Pt device. Only one semicircular arc, which was assigned to the bulk component, was observed in the Cole-Cole plots. The decrease in the diameter of the semicircular arc was observed by switching from the high to low resistance states. The change in the bulk component corresponds to the overall resistance change in the Ni/PCMO/Pt device.

**Figure 7:**
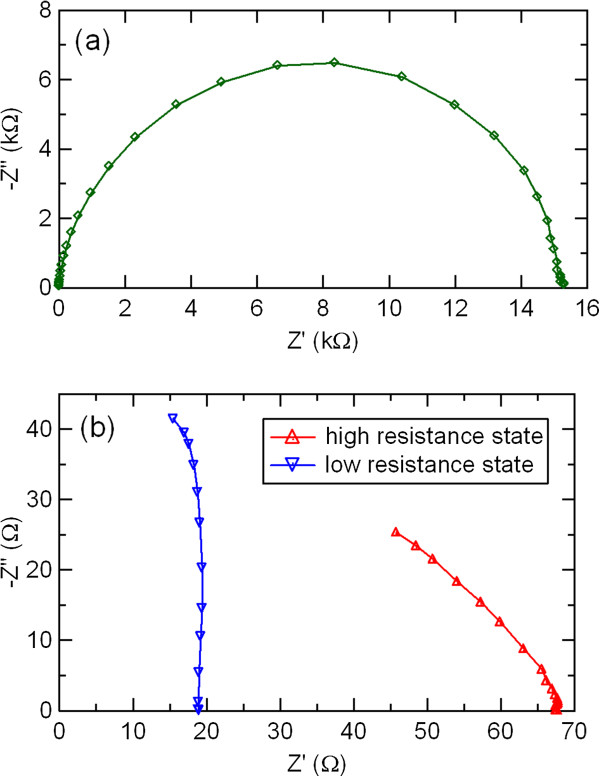
**Impedance spectra of (a) initial state and (b) high and low resistance states in the Ag/PCMO/Pt device.** The solid line connects experimental data points.

Figure 7 shows impedance spectra of the initial, high resistance, and low resistance states in the Ag/PCMO/Pt device. Only the structure due to the bulk component of these three states was observed in the Cole-Cole plots. The resistance in the high and low resistance states was smaller than that in the initial state. A part of a semicircular arc was observed in the high and low resistance states, while a complete semicircular arc was seen in the initial state. The change in the bulk component was detected by applying an electric voltage for resistance switching.

The real part of impedance at 0 Hz measured by alternating current (ac) impedance spectroscopy corresponds to the dc resistance of the device. On the contrary, the real part values of impedance at 0 Hz shown in the impedance spectra (Figures 5, 6, and 7) do not show a good agreement with the resistance values shown in the electric-pulse-induced resistance switching behavior (Figures 1b, 2, and 3b, respectively). The same top electrode material and the same characterization technique reproducibly resulted in the similar resistance change. However, the results strongly depend on the techniques. The reason, which is not clear yet, may lie in some intrinsic difference of resistance transition processes between each technique.

**Figure 8:**
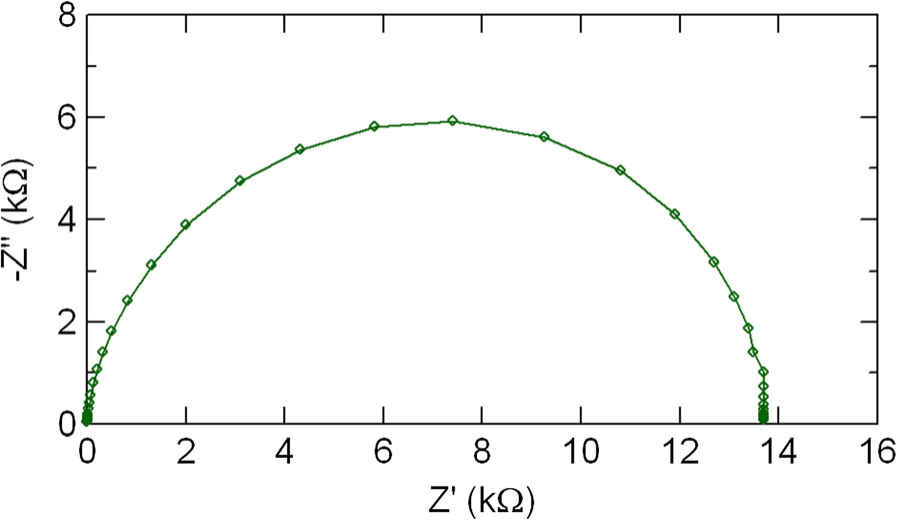
**Impedance spectra of the Au/PCMO/Pt device.** The solid line connects experimental data points.

Figure 8 shows impedance spectra of the Au/PCMO/Pt device. Only one semicircle was observed in the Cole-Cole plot. No change by applying an electric pulse was observed in the Cole-Cole plot.

**Figure 9:**
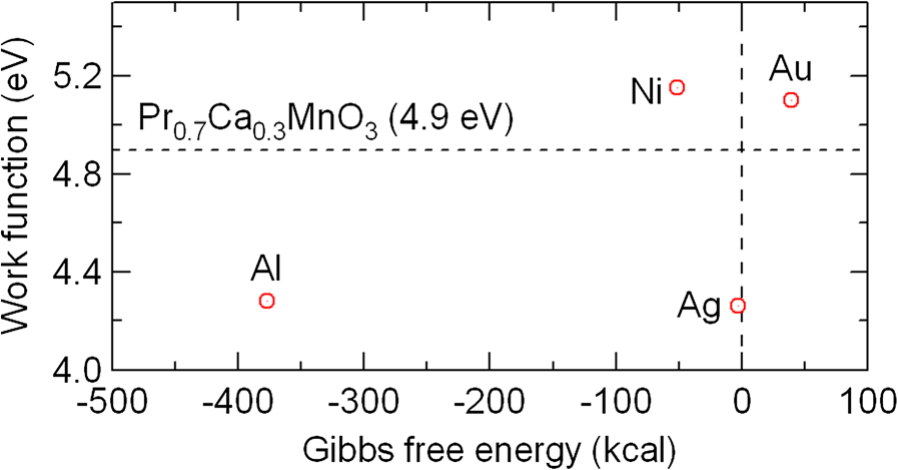
Work function and standard Gibbs free energy of formation of metal oxides of electrode metals.

The work function of the electrode metals is shown in Figure 9. In general, PCMO is a p-type semiconductor with a work function of 4.9 eV [40]. Because Ni and Au have a larger work function than PCMO, a Schottky barrier is not expected to be formed between the top electrode and PCMO in the Ni/PCMO/Pt and Au/PCMO/Pt devices. However, resistance switching was observed in the Ni/PCMO/Pt device (see Figure 2c), while no resistance change was detected in the Au/PCMO/Pt device (see Figure 4b). On the contrary, a Schottky barrier is expected to be formed between the top electrode and PCMO in the Al/PCMO/Pt and Ag/PCMO/Pt devices because the work function of Al and Ag is smaller than that of PCMO. Even though Ag has a similar work function to Al, the resistance switching ratio in the Ag/PCMO/Pt device is much smaller than that in the Al/PCMO/Pt device. The work function is probably not the only cause of the large resistance switching of the Al/PCMO/Pt device.

The standard Gibbs free energy of the formation of metal oxides is also shown together with the work function of the electrode metals in Figure 9. The difference in the oxidation Gibbs free energy between Al and Ag shows a good correspondence with the difference in the resistance switching behavior between the Al/PCMO/Pt and Ag/PCMO/Pt devices. An applied electric field may enhance the oxidation at the interface with the electrode metals with lower oxidation Gibbs free energy. The oxidation near the interface plays a role in the electrical hysteresis and resistance switching. The opposite switching polarity of the Ag/PCMO/Pt device to the Al/PCMO/Pt device is due to the difference in the oxidation Gibbs free energy [41].

As stated above, the resistance switching behavior was significantly different between the Ni/PCMO/Pt and Au/PCMO/Pt devices, although Au has a similar work function to Ni. This difference in the resistance switching also can be explained well by the difference in the oxidation Gibbs free energy between Ni and Au. Whether resistance switching can be observed or not seems to be dependent on the oxidation Gibbs free energy.

Recently, an amorphous Al oxide layer with the thickness of several nanometers was found at the Al/PCMO interface by high-resolution transmission electron microscopy (HRTEM) [18]. It was also reported that the oxidation of Al metal in PCMO films at the Al/PCMO interface was observed by X-ray photoemission spectroscopy (XPS) [19,20]. In order to evaluate the capacitance due to the Al oxide layer at the Al/PCMO interface, the observed impedance spectra shown in Figure 5 were analyzed by comparing with the simulated spectra constructed on the basis of an equivalent circuit composed of parallel connection of resistance and capacitance (RC). Three sets of parallel *RC* components in series were required as an equivalent circuit to reproduce the observed spectra by theoretical simulation, although the experimental impedance spectra seemed to be composed of two semicircular arcs [30]. These three components can be assigned to grain bulk, grain boundary, and film-electrode interface. By fitting the experimental impedance spectra with the simulated ones, the interface resistance values of high and low resistance states were evaluated to be 915 and 15 kΩ, respectively. Simultaneously, the interface capacitance values of high and low resistance states were determined to be 2.5 × 10^−9^ and 7 × 10^−9^ F as the best-fit parameters, respectively. Knowing the interface capacitance *C*, the thickness of the Al oxide interfacial layer, *d* = *ε*_0_*εS* / *C*, can be estimated, where *ε*_0_, *ε*, and *S* are the vacuum permittivity, the dielectric constant of aluminum oxide, and the electrode area, respectively [33]. With *ε*_0_ = 8.85 × 10^−14^ F/cm, *ε* = 10, and *S* = 2 × 10^−3^ cm^2^, *d* is obtained to be 7 and 2.5 nm in the high and low resistance states, respectively. The thickness of the Al oxide interfacial layer obtained by impedance spectroscopy in this work was in good agreement with that estimated by HRTEM and XPS [18-20]. The oxidation of the Al electrode plays a dominant role in the bipolar resistance switching in the PCMO-based devices. On the contrary, the resistance change at the interface might not give a dominant contribution to the overall resistance change of Ni/PCMO/Pt and Ag/PCMO/Pt devices because with Ni and Ag, it is difficult to form the oxide interface layer as compared with Al. As a result, the resistance change ratio of Ni/PCMO/Pt and Ag/PCMO/Pt devices is smaller than that of the Al/PCMO/Pt device. It is rather difficult to categorize Ni and Ag into the group of top electrode materials that cause the ReRAM effect.

## Conclusions

The electric-pulse-induced resistance switching in manganite film-based devices with various metal electrodes of Al, Ni, Ag, and Au was studied by dc current–voltage measurements and ac impedance spectroscopy. The hysteretic *I-V* characteristics and resistance switching were observed in the PCMO-based devices with top electrode of Al, Ni, and Ag. The Al/PCMO/Pt device showed larger resistance switching than other PCMO-based devices with top electrode of Ni and Ag. The electrode material dependence of the resistance switching in polycrystalline manganite films was investigated in more detail by impedance spectroscopy. Two semicircular arcs were observed in the impedance spectra of the Al/PCMO/Pt device, while the Cole-Cole plots in the devices with Ni, Ag, and Au showed only one semicircular arc. These two distinctive features of the Al/PCMO/Pt device could be assigned to the PCMO bulk and to the interface between the PCMO film and the Al electrode, respectively. By comparing the impedance spectra between the high and low resistance states in the Al/PCMO/Pt device, we suggested that the resistance switching in the PCMO-based devices was mainly due to the resistance change in the interface between the film and the electrode. According to the theoretical simulation of impedance spectra, the interface component observed by impedance spectroscopy in the Al/PCMO/Pt device might be due to Al oxide layer formed by oxidation of Al top electrode. The interfacial transition layer of Al oxides is possibly responsible for the large resistance change in the Al/PCMO/Pt device. The oxidation Gibbs free energy rather than work function gives dominant influence on the resistance switching behavior of the PCMO-based devices. Large resistance switching ratio is expected by choosing a metal with lower oxidation Gibbs free energy as an electrode material and using the interface resistance component due to metal oxide layer in the PCMO-based devices.

## Competing interests

The authors declare that they have no competing interests.

## Authors’ contributions

TN designed this study and carried out the experiments. KH performed the experiments under the guidance of TN. KT participated in the coordination of the study. All authors discussed the results. TN wrote the manuscript. All authors read and approved the final manuscript.
